# Molecular subtypes in Alzheimer's disease ‐ transcriptome and beyond

**DOI:** 10.1002/alz70855_096029

**Published:** 2025-12-23

**Authors:** Bin Zhang

**Affiliations:** ^1^ Department of Genetics and Genomic Sciences, Icahn School of Medicine at Mount Sinai, New York, NY, USA; Icahn Genomics Institute, Icahn School of Medicine at Mount Sinai, New York, NY, USA; Icahn School of Medicine at Mount Sinai, New York, NY, USA; Mount Sinai Center for Transformative Disease Modeling, Icahn School of Medicine at Mount Sinai, New York, NY, USA; Ronald M. Loeb Center for Alzheimer's Disease, New York, NY, USA; Mount Sinai Center for Transformative Disease Modeling, New York, NY, USA

## Abstract

**Background:**

Late‐onset Alzheimer's disease (LOAD), the most common form of dementia, is driven by a complex interplay of genetic, epigenetic, and environmental factors, resulting in considerable heterogeneity in disease mechanisms. This variability poses a major challenge in developing effective therapies for the broader LOAD patient population.

**Method:**

We conducted sample clustering analyses to identify molecular subtypes of LOAD using transcriptomic, proteomic, and methylomic data. Differential expression analysis was then used to uncover the molecular signatures associated with each subtype. We further applied drug repurposing approaches to predict potential therapeutic candidates tailored to each AD subtype.

**Result:**

We have recently identified five transcriptomic subtypes of LOAD across multiple large, independent cohort studies. Each subtype exhibits a distinct set of dysregulated genes. Additionally, we have identified two corresponding subtypes in proteomic and DNA methylation data, which align with the atypical and typical transcriptomic subtypes of LOAD. The discovery of these robust molecular subtypes has prompted the development of novel therapeutic strategies for treating LOAD, including the repurposing of FDA‐approved or experimental drugs tailored to each subtype. Several of our top repurposed drug candidates have already been experimentally validated. These preliminary findings demonstrate that our innovative drug discovery framework has the potential to significantly accelerate the identification of effective treatments for LOAD, while dramatically reducing the risks and costs.

**Conclusion:**

Integrating disease subtype analysis of LOAD with drug repurposing presents a novel and promising strategy for delivering targeted treatments based on the unique molecular profiles of more homogeneous patient subgroups, laying the foundation for precision medicine in Alzheimer's disease.

